# AI-Enhanced Transcriptomic Discovery of Druggable Targets and Repurposed Therapies for Huntington’s Disease

**DOI:** 10.3390/brainsci15080865

**Published:** 2025-08-14

**Authors:** Rodrigo Pinheiro Araldi, João Rafael Dias Pinto, Irina Kerkis

**Affiliations:** 1 BioDecision Analytics Ltd., São Paulo 04021-001, Brazil or rodrigo.araldi@unifesp.br (R.P.A.); joao.dias@biodecisionanalytics.com (J.R.D.P.); 2 Structural and Functional Post-Graduation Program, Paulista School of Medicine, Federal University of São Paulo (UNIFESP), São Paulo 04021-001, Brazil; 3 Genetics Laboratory, Butantan Institute, São Paulo 05503-900, Brazil

**Keywords:** Huntington’s disease, BDASeq^®^, drug repurpose, neurodegenerative disorder, physiopathology, molecular pathology, treatments, pharmacogenomics, differential expression analysis

## Abstract

Huntington’s disease (HD) is an autosomal dominant neurodegenerative disorder characterized by progressive motor dysfunction, psychiatric disturbances, and cognitive decline. The pathophysiology of HD centers on a polyglutamine expansion in the huntingtin protein, which triggers widespread transcriptional dysregulation, impaired proteostasis, mitochondrial dysfunction, and excitotoxic neuronal loss—most prominently within the striatum and cortex. Despite decades of research, disease-modifying therapies remain elusive. This review synthesizes how the emerging integration of translational bioinformatics, spotlighting artificial intelligence-driven transcriptomic analyses, has identified transcriptional signatures correlating with disease progression and therapeutic response. These integrative approaches hold promise for accelerating the bench-to-bedside translation of HD therapeutics, positioning AI-powered discovery as a frontier for overcoming the complexity of neurodegeneration.

## 1. Huntington’s Disease: From the First Clinical Report to the Discovery of Its Etiology

Huntington’s disease (HD, OMIM 143100) is a rare and fatal monogenic autosomal-dominant neurodegenerative disorder characterized by progressive motor dysfunction, neuropsychiatric symptoms, and cognitive decline [1,2].

Although HD prevalence varies considerably across geographic regions, global estimates suggest it affects approximately 4.88 individuals per 100,000 population [1,2]. Notably the prevalence is higher in populations of European (6.37 per 100,000; 95% CI, 4.50–8.81) and North American ancestry (8.87 per 100,000; 95% CI, 4.69–16.78) [1]. The elevated prevalence is attributed to several factors, including the ability of individuals with HD gene mutation to pass it on to the next generation, improved diagnostic molecular tools, and the reclassification of previously misdiagnosed cases [3,4,5]. In contrast, much lower prevalence is reported in regions such as Africa (0.25 per 100,000) and East Asia (0.41 per 100,000), likely reflecting differences in genetic background, healthcare infrastructure, and diagnostic practices.

In Brazil, HD remains underdiagnosed, with limited nationwide epidemiological data available. The principal source of such data is the registry maintained by the Brazilian Huntington Association (ABH), founded in 1997. According to ABH estimates, approximately 14,000–20,000 individuals currently live with HD, while 73,000–100,000 are at risk of developing the disease. Interestingly, some regions in Brazil exhibit a disproportionately high prevalence of HD, a phenomenon attributed primarily to genetic clustering within isolated populations. Notably, cities such as Feira Grande (Alagoas), Senador Sá (Ceará), and Ervália (Minas Gerais) have been identified as HD hotspots, with Feira Grande reporting a prevalence of 10.4 per 10,000 inhabitants—significantly above the national average [4]. These elevated rates are likely the result of founder effects, where a small number of ancestors carrying the mutant HTT gene contributed disproportionately to the genetic makeup of subsequent generations. Limited genetic diversity and consanguinity in these communities may have amplified the transmission of the disease.

The clinical presentation of HD varies among individuals, but typically affects motor, neuropsychiatric and cognitive domains, with symptoms severity increasing over time [5,6,7].

In the early stage, patients may present subtle involuntary movements such as chorea, clumsiness, or impaired coordination (motor symptoms). Psychiatric symptoms often include depression, irritability, and anxiety, apathy, or obsessive–compulsive behaviors. Cognitive manifestation commonly involves difficulties with multitasking, slower processing speed, and impaired organizational or planning abilities [8].

As the disease progresses to the middle stage, choreic movements become more pronounced and may involve the limbs and facial muscles. Voluntary motor functions such as ambulation and speech are increasingly compromised. Psychiatric manifestation may worsen, with mood swings, impulsivity, and symptoms of mania or severe depression. Social behavior may be disrupted due to disinhibition or irritability, and cognitive deficits—particularly in memory, reasoning, and problem-solving—may become more evident [8].

In the late stage, chorea typically diminishes, while rigidity and bradykinesia (slowness of movements) predominate. Swallowing difficulties and severe motor impairment often necessitate full-time care. Profound apathy and social withdrawal are frequently observed. In some cases, psychosis and delusional thinking emerge. Dementia develops, severely impairing communication, memory, and comprehension [8].

**Figure 1:**
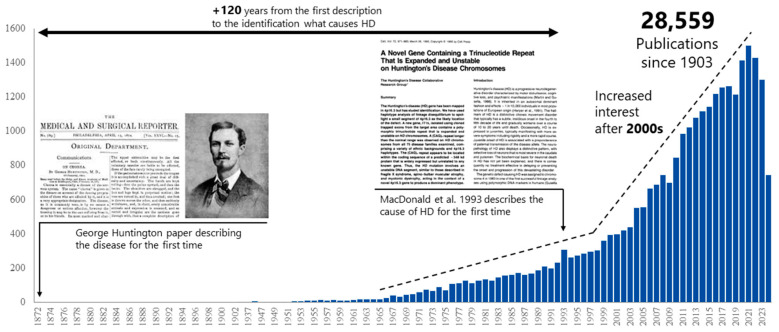
Growth in Huntington’s disease research publications over time. Number of publications related to Huntington’s disease indexed in PubMed from the initial clinical description in 1872 to 10 March 2025. A marked increase in publication volume is observed beginning in 1993, coinciding with the discovery of the genetic cause of HD—the CAG trinucleotide repeat expansion in the *HTT* gene—highlighting the pivotal impact of this breakthrough on research activity in the field [11].

HD was first described in 1872 by George Huntington in the seminal publication “On chorea”, where he characterized a hereditary disorder marked by involuntary movement, cognitive impairments, and psychiatric disturbances, as observed in families from Long Island, New York [9,10]. However, the genetic basis of the disease remained elusive for more than a century. In 1993, Huntington’s Disease Collaborative Research Group identified the casual mutation: an expansion of cytosine-adenine-guanine (CAG) trinucleotide (>36) repeats in the first exon of the *HTT* gene, located at chromosome 4p16.3. This gene encodes the huntingtin protein (HTT) [11]. Since Huntington’s initial report, over 28,000 scientific articles on HD have been published, with the vast majority in the past two decades (Figure 1), reflecting a growing global interest in understanding and treating this devastating disease.

## 2. How Is the CAG Expansion Involved in HD Physiopathology?

In the general population, the wild-type *HTT* allele (wt*HTT*) typically contains fewer than 36 CAG repeats, most commonly ranging from 10 to 26 [12]. About 6% of healthy people have intermediate alleles (27–35 CAG repeats), which do not cause disease but can be unstable during germline transmission and may expand to pathogenic ranges in future generations [13,14,15]. Interestingly, the frequency of IAs varies considerably across populations, from 0.45% in Thailand to 8.7% in Brazil [13].

Although IA carriers are not formally diagnosed with HD, a subset of individuals with intermediate repeat lengths have been reported to exhibit HD-compatible phenotype, including movement disorder, cognitive decline, and neuropsychiatric disturbances [13,15,16,17,18,19,20].

**Figure 2:**
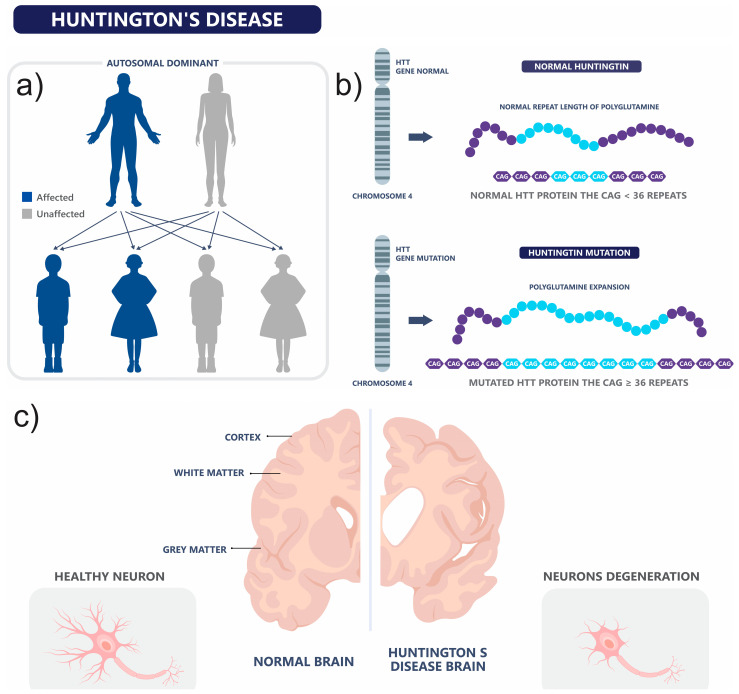
Genetic etiology and pathophysiological consequences of HD. Schematic representation of the genetic basis of HD. (**a**) HD is an autosomal dominant disorder caused by a CAG trinucleotide repeat expansion in the first exon of the *HTT* gene. (**b**) This expansion encodes an abnormally elongated polyglutamine (polyQ) tract in the huntingtin protein. Individuals with intermediate alleles (27–35 CAG repeats) are typically unaffected but may transmit expanded alleles into the pathogenic range during germline transmission. Some carriers of intermediate alleles can exhibit HD-like phenotypes, including motor dysfunction, cognitive decline, and behavioral disturbances. Individuals with ≥36 CAG repeats are considered mutation carriers: those with 36–39 repeats show reduced penetrance and may remain asymptomatic, whereas those with ≥40 repeats exhibit full penetrance and develop clinical HD. (**c**) The mutant huntingtin protein (mHTT) induces widespread cellular dysfunction, ultimately leading to neuronal degeneration, with prominent loss observed in the striatum.

In contrast, individuals harboring mutated *HTT* allele (mHTT), characterized by more than 36 CAG repeats, are at risk of developing HD. The pathogenic threshold is stratified into reduced full penetrance categories: alleles with 36–39 CAG repeats are associated with reduced penetrance, meaning some carriers may remain asymptomatic throughout life, while alleles with ≥40 repeats confer full penetrance and are invariably associated with clinical manifestation of the disease [21], as summarized in Figure 2.

Huntingtin (HTT) is a soluble α-helical protein with a molecular weight 348 kDa, which is composed of 3144 amino acids and is encoded by the *HTT* gene [22]. Although it is ubiquitously expressed, HTT is found to have the highest concentration in the central nervous system (CNS), where it plays a crucial role in both CNS development and homeostatic maintenance [7,23]. This is supported by studies showing that *HTT*-null embryos (wt*HTT*-/-) exhibit developmental delay and disorganization, leading to death between embryonic days 8.5 and 10.5 [24,25,26]. In contrast, *HTT*-heterozygous mice (wt*HTT*+/-) develop motor and cognitive impairments, along with significant neuronal loss—up to 50% in the *globus pallidus* and the subthalamic nucleus [26,27,28], although no cell death is observed in the striatum, the primary brain area affected by HD [28]. These effects are attributed to the HTT protein’s interaction with over 200 other proteins, participating in numerous cellular and biological processes, including vesicular transport and recycling, endocytosis, endosomal trafficking, autophagy, and transcription regulation, the cell cycle, and energy metabolism—functions that are particularly important for neuronal synaptic activity [7,12,28].

Biochemically, the N-terminal region of HTT is the most extensively studied region, as it contains (polyQ) tract encoded by the CAG repeats implicated in HD [7].

**Figure 3:**
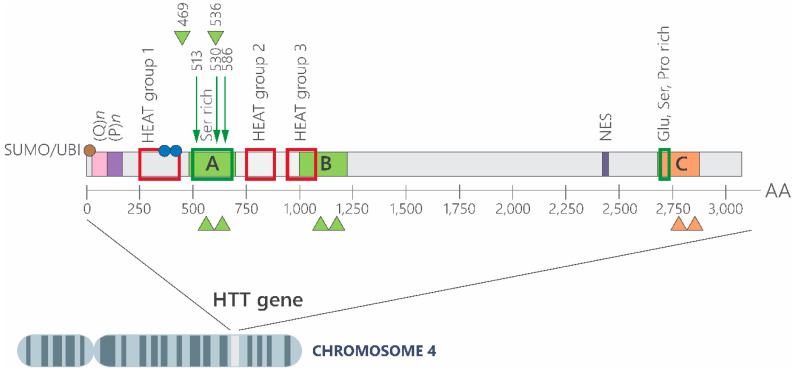
Structural features and proteolytic processing sites of the huntingtin protein. Schematic representation of the huntingtin amino acid sequence. The polyglutamine tract, denoted by (Q)ₙ, is followed by a proline-rich domain (P)ₙ. Red squares indicate the three main clusters of HEAT repeats, which contribute to the protein’s scaffold-like architecture. Arrows denote caspase cleavage sites and their corresponding amino acid positions, while blue arrowheads indicate calpain cleavage sites. Proteolytic regions preferentially cleaved in the cortex (B), striatum (C), or both regions (A) are labeled accordingly. Green and orange arrowheads mark additional protease-sensitive regions. The nuclear export signal (NES) is also indicated. Red and blue circles represent post-translational modifications: red denotes sites of ubiquitination (UBI) and/or sumoylation (SUMO); blue marks phosphorylation sites at serine 421 and serine 434. Glutamic acid (Glu)-, serine (Ser)-, and proline (Pro)-rich regions are shown, with serine-rich domains highlighted by green outlines. These structural and regulatory elements underscore the complex post-translational modulation and proteolytic fragmentation that contribute to huntingtin’s pathogenicity. Image adapted from Cattaneo et al. [39].

The polyQ sequence beginning at residue 18 typically comprises up to 34 glutamines in unaffected individuals. It is preceded by 17 conserved residues (N17) essential for endoplasmic reticulum retentions [8,29,30,31], and it is followed by a proline-rich domain (PRD) [8], which mediates interaction with proteins that contain tryptophan in the Src homolog 3 (SH3) domains [29,32]. The remaining 97% of the protein, encoded by exon 2–67 (amino acid 69-3144), is less well characterized but includes HEAT repeats involved in scaffolding multiprotein complexes [33,34,35] and the nuclear export signal (NES), subject to post-translational modifications such as acetylation, sumoylation, and ubiquitination [36,37]. HTT also contains multiple proteolytic cleavage sites; both wild type and mHTT can be cleaved by caspases (e.g., caspase-3 at residues 469, 513 and 536, and 552; caspase-1 at 572) and the metalloproteinase (MMP)-10 at residue 402 [38], as shown in Figure 3.

The expansion of CAG trinucleotide in HTT leads to increased polyQ residues, reducing solubility of mHTT protein and promoting intracellular aggregates (inclusion bodies), particularly in GABAergic medium spiny neurons (MSNs) of the striatum that express dopamine D2 receptor (D2R), DARP32, and enkephalin [7,40]. MSNs constitute 85–95% of striatal neurons and are essential for motor control and higher-order cognitive functions, such as reward processing and decision-making [41,42].

Initially, insoluble mHTT proteins were thought to drive neurodegeneration [41,42,43,44,45]. However, evidence showing accumulation of toxic N-terminal mHTT fragments in various neuronal compartments suggested the cleavage products may be more pathogenic, while larger aggregates may serve a protective sequestration role [12,44,46,47,48,49]. The precise contribution of these aggregates remains debated. Notably, N-terminal mHTT fragments impair mitochondrial axonal transport even in the absence of inclusion bodies, indicating an early pathogenic mechanism [50].

Moreover, mHTT undergoes differential ubiquitination: K48-linked ubiquitination promotes degradation, while K63-linked ubiquitination promotes aggregation. The age-related decline of Ube3a, essential for K48-linked ubiquitination, may explain symptom onset in mid-life and IB accumulation in late-stage HD [51,52].

Since the discovery of mHTT in 1993, it has been implicated in a cascade of pathogenic events, including mitochondrial dysfunction [2,53], metal ion dyshomeostasis [54,55,56], cholesterol imbalance [57,58,59], oxidative stress [7,60,61], excitotoxicity [62,63], impaired proteostasis [64,65], and neuroinflammation [66,67]. This review summarizes the mHTT role in each of these interconnected processes.

### 2.1. mHTT Contributes to Mitochondrial Dysfunction and Increased Oxidative Stress

Neurons require a continuous energy supply to maintain their complex functions, with mitochondria playing a central role by generating adenosine triphosphate (ATP) through oxidative phosphorylation [68,69]. This process converts acetyl coenzyme A (Ac-CoA)—derived from carbohydrates, fatty acids, and amino acids—into ATP with high efficiency, yielding over 30 ATP per glucose, in contrast to only 2 from glycolysis [68,70,71]. Due to limited ATP storage capacity, sustained mitochondrial activity is critical for cellular homeostasis [68].

However, oxidative phosphorylation is also a primary endogenous source of reactive oxygen species (ROS). During electron transport, a fraction of electrons prematurely reacts with molecular oxygen, forming superoxide anion (O_2_^−^) [72].

These reactive oxygen species can damage cellular components, contributing to oxidative stress—a key feature of mHTT-induced mitochondrial dysfunction in HD.

Superoxide is a highly reactive free radical containing an unpaired electron, enabling it to donate or accept electrons from biomolecules, such as proteins, lipids, metabolites and even nucleic acids, leading to structural damage or functional disruption. To limit this toxicity, superoxide is rapidly converted into less reactive hydrogen peroxide (H_2_O_2_) by superoxide dismutase SOD1 (cytosolic) or SOD2 (mitochondrial) through a reaction often linked to Fenton chemistry [73,74].

Although less reactive than superoxide, hydrogen peroxide can still damage biomolecules. To prevent this, glutathione peroxidase (Gpx) reduces H_2_O_2_ to water using reduced glutathione (GSH) as an electron donor [75]. The mHTT interacts with mitochondrial protein AIFM1, impairing complex I (NADH oxidase) activity and leading to OXOPHOS deficiency and increased reactive oxygen species production, as complex I is a major source of superoxide [76,77,78]. Additionally, mHTT selectively reduced complex II activity, particularly in striatal MSNs [74]. Restoration of complex II function via ectopic expression of its subunits rescue mitochondrial activity and cell viability [79,80]. Supporting this, exposure to 3-nitro propionic acid (3-NP), a selective complex II inhibitor, reproduces MSN loss and HD-like motor symptoms in rodents and humans—though chorea is typically absent in animal models [56,61,81,82,83,84].

Nuclear mHTT aggregates also sequester transcriptional factors, disrupting antioxidant gene expression [84,85], including genes regulated by peroxisome proliferator-activated receptor gamma coactivator 1-alpha (PGC-1α), a master coactivator of mitochondrial biogenesis and antioxidant defense [77,86,87,88,89]. In HD, mHTT reduces PGC-1a levels directly and via transglutaminase (Tgase) activation [61,84,85], lowering the expression of antioxidant enzymes such as SOD-1, SOD-2, and Gpx-1 [77,84,86]. These alterations lead to mitochondrial dysfunction, elevated reactive oxygen species, and bioenergetic failure, contributing to neurodegeneration [87].

Although oxidative stress is consistently observed in HD [88,89], clinical trials using classical antioxidants have failed to show therapeutic benefits [90,91,92,93,94]. This may reflect uncertainly over whether oxidative stress is a cause or a downstream effect of primary HD mechanism such as transcriptional and mitochondrial dysfunction [72,95,96,97,98].

### 2.2. Mutant Huntingtin (HTT) Protein Leads to Dysregulation of Metal Homeostasis

Mutated HTT disrupts metal and calcium homeostasis, both contributing to mitochondrial dysfunction. It enhances mitochondrial Ca^2+^ uptake NMDA receptor-mediated influx, increasing mitochondrial ROS and mitochondrial DNA (mtDNA) damage [99,100]. Additionally, mHTT induces abnormal release of transition metals (Cu, Fe, Zn) from their storage sites, promoting oxidative stress [101,102,103,104].

Iron (Fe) and copper (Cu) are particularly implicated in HD, with elevated levels found in HD postmortem patient brains, R6/2 mice, and in a *Drosophila* model [54,99,100]. Magnetic resonance image (MRI) studies confirmed iron accumulation in the basal ganglia and cortex of HD patients, correlating with CAG repeat length and disease severity [54]. However, the regulation of metal transporters such as transferrin receptor, ferroportin, and metallothionein remains poorly characterized in HD [56,105].

Iron contributes to oxidative damage primarily via Fenton chemistry and activation of iron dependent enzymes like hypoxia inducible factor (HIF) prolyl hydroxylases [54,106]. Copper, in turn, binds directly to the N-terminal domain of HT, catalyzing cysteine oxidation and promoting toxic oligomer formation due to impaired autophagic clearance [107,108,109]. Copper can also exacerbate reactive oxygen species production through redox cycling.

Metal chelators have been explored as therapeutic agents, but most high-affinity compounds exhibit limited blood–brain barrier permeability due to high weight (>500) or hydrophilicity [110]. For example, intraventricular administration of deferoxamine (DFO) improved motor behavior in R6/2 mice [105], yet systemic DFO (MW = 657) is unlikely cross the BBB effectively, limiting clinical utility [111].

### 2.3. mHTT and Its Role in Excitotoxicity

Excitotoxicity, characterized by excessive glutamate-induced activation of NMDA receptors, contributes to neuronal death in HD [112,113,114]. NMDA receptors are formed by NR1, NR2 (A–D) and NR3 (A–B) subunits [62]. In vitro studies using HEK293 cells co-expressing mHTT and NMDA receptor subtypes revealed that mHTT selectively induces apoptosis in NR1/NR2B expressing cells [115], aligning with the vulnerability of neostriatal MSNs, where NR2B predominates [62,115,116].

Moreover, mHTT promotes aberrant extrasynaptic glutamatergic signaling and mislocalization of NMDARs, disrupting the CREB-PGC-1α pathway [116]. As PGC-1α regulates mitochondrial antioxidant defenses [117], its dysfunction contributes to oxidative stress and neuronal damage [112].

### 2.4. Mutated HTT Promotes Cholesterol Metabolism Deregulation

Despite representing only ~2% of total body mass, the CNS contains the highest cholesterol average concentration (15–20 mg/g) for other tissues, largely unesterified and concentrated in oligodendrocyte-derived myelin [113,114]. Cholesterol plays critical roles in membrane organization, receptor function, and synaptic vesicle dynamics [118,119,120,121,122,123,124,125,126]. Its depletion destabilizes lipid rafts, progressively impairing synaptic integrity and neuronal viability [125,126]. Although cholesterol turnover in the adult CNS is remarkably slow, disturbances in cholesterol homeostasis have been linked to neurodegenerative disorders, including HD [113]. In HD models and postmortem brains, the biosynthesis of cholesterol and fatty acids is consistently impaired [123,127,128] due to the reduced activity of sterol regulatory element-binding proteins—SREBP-2 for cholesterol and SREBP-1c—for fatty acid [113]. This dysregulation leads to diminished transcription of key biosynthetic enzymes, such as HMG-CoA reductase (for cholesterol synthesis) and fatty acid synthase (FASN) (for fatty acid synthesis), contributing to early pathogenic changes.

A downstream consequence is the decreased concentration of 24S-hydroxycholesterol (24-OH-Chol), a brain-derived metabolite that crosses the blood–brain barrier and may serve as a surrogate biomarker of cholesterol synthesis and disease progression [129,130]. Supporting this, HD patients exhibit a plasma cholesterol level ~40mç/dL lower than that of healthy individuals [113,131]. Although CNS-blood cholesterol correlation is limited, 24-OH-Chol accounts for ~10% of total plasma cholesterol [132].

Fatty acid metabolism is similarly affected due to shared transcriptional regulation via SREBPs [113,133]. HD patient-derived fibroblasts exhibit reduced growth under lipid-depleted conditions, reversible by linoleic and linolenic acids supplementation [113,132]. Moreover, altered membrane fluidity observed in HD cells is consistent with aberrant fatty acid elongation and desaturation [114,134].

Palmitoylation—a reversible lipid modification that facilitates membrane targeting and intracellular trafficking—is disrupted in HD. This is attributed to impaired interaction between huntingtin and its palmitoyltransferase, the huntingtin-interacting protein, particularly in striatal neurons [114,135,136]. Loss of palmitoylation comprises huntingtin’s subcellular localization and neuronal transport, contributing to dysfunction [137].

Despite increased appetite and caloric intake, HD patients frequently loss weight due to hypercatabolic state marked by elevated resting energy expenditure and altered fatty tissue and acid metabolism [40,138]. The mechanism underlying this energy imbalance remain poorly understood but are associated with disease progression.

Moreover, mHTT also suppress brain-derived neurotrophic factor (BDNF) transcription and vesicular transport [40,127,128,129,130,139,140,141,142,143], compounding metabolic deficit. BDNF plays a critical role in cholesterol biosynthesis and lipid exchange between neurons and glia [138]. Its deficiency in HD contributes to cholesterol scarcity, exacerbating neuronal vulnerability. Therapeutic strategies aimed at restoring BDNF signaling have shown promise, normalizing lipid metabolism and preserving neuronal function in preclinical HD models [40,114,130,135,136,140,144,145,146,147,148].

### 2.5. Systemic Effects of Mutated Huntingtin

**Figure 4:**
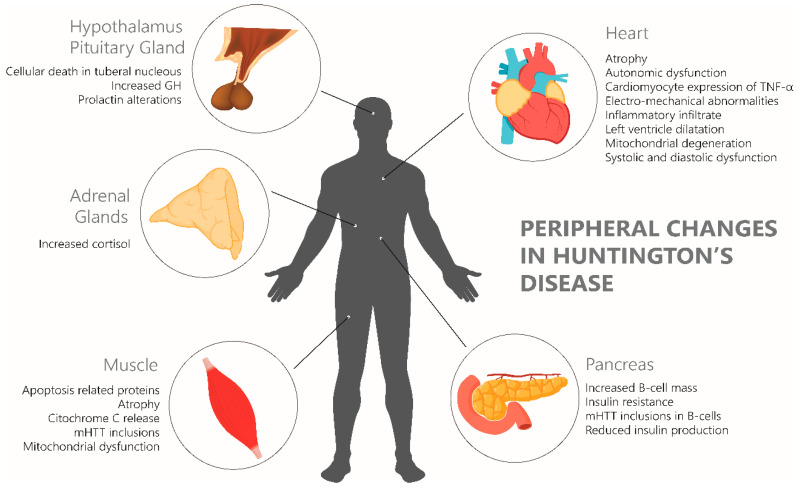
Peripheral organ involvement in HD. Schematic illustration highlighting extra-neuronal manifestations of HD. Mutant huntingtin expression perturbs multiple peripheral tissues, including striated skeletal muscle (myopathy and altered energy metabolism), striated cardiac muscle (cardiomyopathy and conduction abnormalities), pancreas (β-cell dysfunction and impaired glucose homeostasis), hypothalamus (disrupted energy balance and neuroendocrine signaling), hypophysis (pituitary hormone dysregulation), and adrenal glands (aberrant stress axis responses). Together, these systemic alterations underscore the multisystemic nature of Huntington’s disease and reinforce the need for therapeutic strategies that address both central and peripheral pathophysiology.

While HD is primarily recognized as a neurodegenerative disorder, mounting evidence highlights its systemic nature, with pathological changes extending beyond the CNS [149,150,151]. The expression of both wild-type and mHTT has been confirmed in multiple peripheral tissues, including skeletal muscle, liver, heart, kidney, pancreas, testis, stomach, and immune cells (Figure 4). This finding supports the concept that HD is a multisystem disorder, with peripheral manifestation contributing to disease progression [150].

Furthermore, mHTT disrupts crosstalk between the brain and peripheral organs [149], contributing to widespread metabolic dysfunction. HD individuals frequently exhibit weight loss despite normal or increased caloric intake, driven by altered lipid and glucose metabolism and peripheral insulin resistance [152,153]. Mutant HTT also promotes oxidative stress and low-grade systemic inflammation, increasing cardiovascular risk [154].

Gastrointestinal disturbances are increasingly reported in HD, including changes in gut motility and microbiome composition [154], further supporting the role of mHTT in peripheral tissue homeostasis. In parallel, hormonal dysregulation, involving cortisol and thyroid hormone, reflects mHTT-induced disruption of endocrine signaling [153]. Moreover, mHTT impairs peripheral neuronal, leading to sensory abnormalities and autonomic dysfunctions such as altered thermoregulation and blood pressure control [154].

These systemic alterations highlight the need for therapeutic strategies that extend beyond CNS targeting. Addressing the peripheral consequences of mHTT may be essential for improving disease outcomes and quality of life in HD.

## 3. Therapeutic Opportunities Targeting HD Pathophysiology

HD has traditionally been viewed as a striatal disorder, primarily Gamma-aminobutyric acid (GABA)ergic neurons in the basal ganglia, particularly those expressing dopamine D1 and D2 receptors [155,156]. These neurons receive convergent glutamatergic inputs from the cortex and dopaminergic projections from substantia nigra, forming cortico-nigrostriatal circuits [157,158]. In addition to excitatory input, cortical glutamatergic neurons are also the principal source of BDNF, which is essential for the survival of MSNs.

Glutamate is the most abundant excitatory neurotransmitter in the CNS, mediating key processes such as synaptic plasticity and learning, including motor cognition [159,160,161]. Approximately 40% of all neurons are glutamatergic, and over 90% of neurons express glutamate receptors, with highest density found in the frontal cortex [162].

Synaptic plasticity—the ability of neurons to adapt their structure and function in response to activity and environmental cues—is often calcium-dependent and relies on dynamic remodeling of the dendritic actin cytoskeleton, as well as localized mRNA translation of synaptic proteins [163,164,165]. Dopamine modulates this plasticity by regulating glutamatergic transmission, dendritic integration, and the intrinsic excitability of MSNs [157,166], thereby contributing to motor control and emotional regulation.

The integrity of cortico-striatal circuits is critical for MSN survival [128], in part because cortical neurons provide BDNF, a pro-survival neurotrophin required for striatal homeostasis [167,168]. In HD, BDNF levels are markedly reduced in both patient’s brains and transgenic animal models [169,170]. This reduction is attributed to impaired transcription of the *BDNF* gene caused by mHTT [169], as well as disrupted axonal transport of BDNF from cortical neurons to the striatum [159].

In addition to cortical glutamatergic neurons, astrocytes also present an important source of BDNF [160,161,163]. However, mHTT disrupts the BDNF in astrocytes through multiple mechanisms, including interference with transcriptional regulation and vesicular trafficking. One key pathway involves the aggregation of mHTT, which impairs the normal function of transcription factors and intracellular signaling cascades essential for BDNF synthesis [160,161,163].

However, mHTT aggregation is rare in astrocytes from HD brains that express endogenous levels of mHTT [165], suggesting an alternative mechanism of BDNF deregulation. Notably, mHTT impairs BDNF secretion by disrupting the Rab3a-GTP/GDP cycle, which is essential for vesicle docking on the astrocyte membrane [171]. mHTT-expressing astrocytes also show a diminished ATP release and reduced docking of ATP-containing dense-core vesicles, potentially linked to mitochondrial dysfunction [172].

The reduction in BDNF is strongly implicated with HD neuropathology, and its restoration ameliorates phenotypes in HD models [173,174,175]. Nonetheless, clinical translation remains limited. Common HD models fail to recapture chorea, and BDNF’s inability to cross the blood–brain barrier [176] limits systemic delivery. Even when administrated directly into the striatum, BDNF improves neuronal function but not survival [173,177], underscoring its limited therapeutic effect as a monotherapy.

Although BDNF modulates MSNs morphology and connectivity [178,179,180], it does not induce formation of fully functional synapses alone [155]. Thus, BDNF-based therapies are unlikely to address the broad motor, cognitive. and neuropsychiatric impairments in HD. This limitation is consistent with the complex molecular landscape of the disease, which, as demonstrated in our transcriptomic analysis, involves the dysregulation of over 13,000 genes. Accordingly, interventions targeting isolated pathways or molecules are unlikely to modify the natural course of HD.

Tetrabenazine (Xenazine^®^), approved by the FDA in 2008 for chorea in HD, offers motor symptom relief but does not halt or prevent or delay neurodegeneration. It is a vesicular monoamine transporter 2 (VMAT2) inhibitor that depletes presynaptic monoamines, particularly dopamine in the brain, which, in turn, affects D2 receptors [181].

Despite its benefits, tetrabenazine is associated with increased risk of depression and suicidality; in a controlled trial, 19% of patients developed depression, and one suicide occurred in the treatment group, with no such event in the placebo group [182]. Common adverse effects include sedation (28%), akathisia (13%), parkinsonism (7%), depression (5.5%), anxiety (4%), fatigue (2%), and diarrhea (2%) [182].

**Figure 5:**
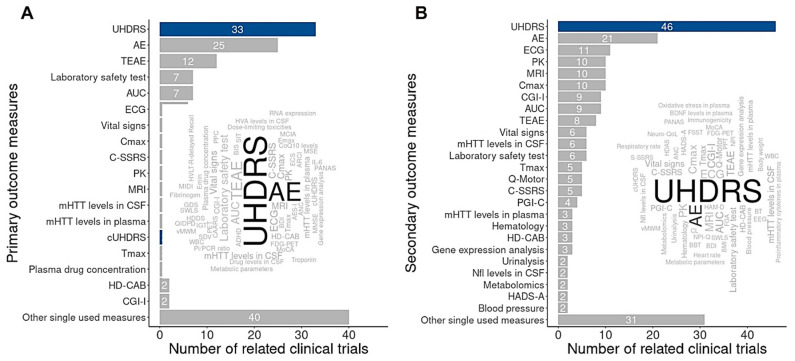
Primary and secondary outcome measures in Huntington’s disease clinical trials. Key clinical features used as outcome measures in trials for HD, categorized into primary (**A**) and secondary endpoints (**B**). Analysis of 256 clinical trials registered on ClinicalTrials.gov as of 1 April 2025 reveals that, in the absence of validated biomarkers, the Unified Huntington’s Disease Rating Scale (UHDRS) remains the most widely used tool to assess treatment efficacy. Data were generated using BioDecision Analytics^TM^ and reflect the current landscape of evidence generation in HD clinical research.

A major barrier to advancing HD therapies remains the lack of robust molecular targets and reliable biomarkers. In the absence of such markers, most of the 256 clinical trials rely on functional scales—primarily the Unified Huntington’s Disease Rating Scale (UHDRS^®^)—as primary or secondary endpoints (Figure 5).

The UHDRS^®^, developed by the Huntington Study Group (HSG), is the most widely used clinical tool to track HD progression across multiple domains, including motor function, cognitive performance, behavioral symptoms, and functional capacity [183]. Given the centrality of motor symptoms in HD, the International Parkinson and Movement Disorder Society commissioned a clinimetric evaluation of six motor rating scales used in HD, including the Abnormal Involuntary Movement Scale, UHDRS-Total Motor Score (UHDRS-TMS), Quantified Neurological Examination, and the Marsden and Quinn Chorea Severity Scale. Among these, only UHDRS-TMS was classified as “recommended” for assessing motor severity based on standardized criteria [184]. Consequently, UHDRS-TMS is the most frequently used primary or secondary endpoint in HD clinical trials. The score (range 0–124) increases at an average rate of ~4.65 points per year (range: 2.9–6.4), although this progression varies according to individual genetic background, disease stage, and comorbid conditions [171].

Another commonly used scale is the UHDRS Total Functional Capacity (TFC), which assesses independence in daily activities, including employment, financial management, domestic chores, and care needs [174]. TFC also enables staging of disease severity: scores of 11–13 typically represent early-stage HD; 7–10 indicate mid-stage; and 0–6 correspond to advanced disease [174,175].

Despite their utility, these scales are limited by the heterogeneity and slow progression of HD symptoms, which reduce sensitivity to change over short observation periods [185]. As a result, there is an urgent need for more robust, objective, and sensitive biomarkers to reliably monitor disease progression and evaluate therapeutic responses in clinical trials.

## 4. Transcriptomic Studies of HD

Multiple approaches have been employed to identify biomarkers that can monitor HD progression and uncover potential therapeutic targets, particularly druggable genes [185]. Given that transcriptional dysregulation is a hallmark of HD, RNA sequencing (RNA-Seq) has emerged as a powerful tool to delineate disease-associated molecular alterations and to guide an attractive the development of biomarkers and therapies [185].

For biomarker discovery, peripheral blood is particularly attractive due to its accessibility and renewable nature [185]. In contrast, the identification of druggable targets required brain tissue. However, using striatal tissue poses challenges due to profound neuronal loss in this region—up to 90% of MSNs are lost in late-stage HD—complicating the acquisition of high-quality transcriptomic data from postmortem striatum. Given the integral role of the cortico-striatal circuity in striatal function physiology, cerebral cortex became an important brain area to be molecularly explored to identify potential drug targets for HD [186,187,188,189].

Given the integral role of the cortico-striatal circuity in maintaining striatal physiology, the cerebral cortex—particularly the Brodmann Area 9 (BA9, dorsolateral pre-frontal cortex)—has become a focal point of transcriptomic investigation [187,188,189,190,191]. Structural MRI studies have demonstrated that BA9 undergoes cortical degradation in HD, including the loss of projection neurons in layers III, V, and VI and increased glial density in deeper layers, particularly layer VI. These findings underscore BA9′s relevance as a surrogate region for molecular profiling and therapeutic target identification.

To date at least four independent transcriptomic studies have investigated brain regions implicated in HD pathology, with a focus on BA9, and the caudate nucleus (CAU) [192,193,194,195,196]. This is because approximately 90% of MSNs are lost in the late stages of the disease [42,192,193,197,198,199]. These efforts, supported by advances in bioinformatics, have enabled in-depth reanalysis and interpretation of gene expression patterns in HD. However, despite these considerable efforts, the differentially expressed genes (DEGs) identified so far have not been translated into clinically actionable biomarkers or viable therapeutic targets [189,191,199,200].

The lack of validated biomarkers and therapeutic targets remains a major bottleneck in HD drug development. In the absence of reliable tools to track disease progression or confirm the efficacy of candidate treatments, the successful translation of preclinical findings into effective therapies continues to be impeded. As a result, no approved disease-modifying therapy currently exists to halt or slow HD progression. Consequently, the burden of patients and their families continues to grow with disease advancement, alongside increasing healthcare and social costs [6].

Economic projections based on a compound annual growth rate (CAGR) of 16.5% estimate that global HD treatment will increase from USD 436 million in 2023 to USD 1728 million by 2032 [201]. This sharp rise underscores the urgent need for effective biomarkers and druggable targets to facilitate the development of therapies that can truly affect the clinical trajectory of HD [6].

## 5. Approved Drugs for HD Treatment

The pharmacological landscape for HD remains markedly limited. To date, three vesicular VMAT2 inhibitors were approved for the treatment of chorea in HD—tetrabenazine (TBZ, *Xenazine^®^*) and deutetrabenazine (DEU, *Austedo^®^*), approved by the U.S. Food and Drug Administration (FDA) on 15 August 2008 and on 3 April 2017, respectively; and Valbenazina (VBZ, *Ingrezza^®^*), approved on August 2023 by the U.S. FDA [194,195,196,202]. These agents act by reducing the presynaptic release of monoamines, particularly dopamine. This mechanism provides relief from chorea, one of the most characteristic motor manifestations of HD. However, these drugs do not influence the underlining neurodegenerative process and thus do not alter the natural course of the disease [194,202,203].

By depleting neuroactive monoamines postsynaptically, these treatments—particularly tetrabenazine and deutetrabenazine—are associated with a high incidence of both common and serious adverse events that may significantly compromise quality of life. Frequently reported adverse effects include sedation, somnolence, and fatigue, all of which may exacerbate the disease’s disabling features. More concerning are severe psychiatric adverse effects, such as depression, anxiety, and dyskinesia, which necessitate treatment discontinuation [181,182,196,202,203,204,205,206]. Of these, depression is particularly significant, occurring in ~19% of tetrabenazine-treated individuals in clinical trial [182], and is strongly associated with increased suicide rick [205]. Consequently, patients receiving these therapies require close psychiatric monitoring throughout the treatment course [207].

In addition to safety concerns, the pharmacokinetic of tetrabenazine presents further clinical challenges. The drug’s short half-life and rapid clearance of its active metabolites result in pronounced plasma concentration fluctuations, necessitating thrice-daily dosing to maintain symptomatic control [203]. These fluctuations are also thought to contribute to CNS-related adverse effects, such as drowsiness, insomnia, and anxiety [203].

Deutetrabenazine, a deuterated form of tetrabenazine, was developed to address these pharmacokinetic shortcomings. The incorporation of deuterium—non-toxic, stable isotope of hydrogen—slows the drug metabolism, extending its half-life and leading to more stable plasma concentration. As a result, deutetrabenazine is associated with a more consistent therapeutic effect and a potentially improved safety profile compared to tetrabenazine [202,203,204].

Although there are no head-to-head clinical trials directly compared tetrabenazine and deutetrabenazine, indirect cooperation through meta-analysis is possible due to the similarity of trial designs between the First-HD study (deutetrabenazine) and the TETRA-HD study (tetrabenazine) [205]. Both agents demonstrated modest reductions in chorea and total motor scores of the UHDRS-TMS relative to placebo (Table 1). Deutetrabenazine was associated with fewer adverse effects than tetrabenazine, though its benefits remained limited to the control of chorea, without broader impact on disease progression.

Similar outcomes were reported in the KINECKT-HD trial evaluating valbenazine, whose mechanism of action remains not completely elucidated [194]. Although the precise mechanism of action of valbenazine in HD remains incompletely understood, it is thought to act through VMAT2 inhibition. Like its predecessors, valbenazine offered only a modest benefit limited to chorea management [194].

Understanding the mechanisms of action and potential adverse effects of available therapies is crucial for optimizing patient outcomes in HD. Given the complexity of these neurodegenerative conditions, distinguishing between symptomatic treatments and those capable of modifying disease biology is essential for guiding clinical decisions. In the absence of disease-modifying therapies, HD management often extends beyond chorea control and induces the use of antipsychotics, antidepressants, and mood stabilizers to address the broader neuropsychiatric spectrum of the disease [208,209,210].

The development of novel therapeutic strategies, especially involving advanced cell-based products, offers a promising path toward altering disease progression. However, the clinical translation of such innovations has proven challenging, and effective disease-modifying treatments remain elusive [7,40,211,212,213,214,215,216,217].

## 6. Translational Bioinformatics and Pharma Intelligence Bring New Horizons for HD Treatment

Given the lack of disease-modifying therapies for HD and the limited efficacy of currently approved drugs, the clinical management of HD often requires the use of multiple medications. These treatments are typically guided by the published literature or clinical experience of practitioners. In this context, drug repositioning—particularly of already approved pharmaceuticals—represents a promising strategy to improve symptom management and overall outcomes in HD patients.

RNA-Seq has emerged as a useful technique for identifying potential druggable genes. These insights can guide rational drug repositioning or inform the development of novel therapeutics though computer-aided drug design (CADD) [218].

CADD is a transformative approach in pharmaceutical research that employs computational methods to streamline drug discovery and development. By integrating biological, chemical, and technological data, CADD helps predict drug behavior, evaluate interactions with biological targets, and optimize pharmacokinetic properties before a compound is synthesized and tested experimentally [218,219].

CADD includes two main strategies: (i) structure-based drug design (SBDD), which uses the 3D structure of a biological target (e.g., a protein) to design molecules capable of binding to it effectively; (ii) ligand-based drug discovery (LBDD), which relies on knowledge of existing molecules that interact with a target to predict new compounds with similar properties [218,219]. These approaches significantly accelerate drug discovery by reducing both time and cost compared with traditional experimental methods. However, the success of CADD depends critically on the prior identification of violable biological targets—namely, druggable genes—highlighting the importance of integrative bioinformatics tools like GNA-Seq in the HD research landscape.

In this regard, RNA-Seq (transcriptomic) analysis is a powerful tool that can significantly contribute to the development of new drugs, including those for HD [189,212,220,221,222]. This technique enables a comprehensive analysis of gene expression patterns, allowing the identification of differences between healthy and HD-affected cells or tissues. RNA-Seq can uncover genes and pathways involved in HD pathophysiology, such as those regulating mitochondrial function, iron metabolism, neuroinflammation, and neurodegeneration [189]. Understanding these mechanisms provides a foundation for targeting specific molecular pathways for therapeutic intervention. Additionally, RNA-Seq has the senility to detect low-abundance transcripts that may be missed by traditional methods, thereby revealing novel drug targets.

The RNA-Seq workflow begins with RNA isolation from samples, followed by either mRNA enrichment or rRNA depletion. For pharmaceutical applications, protein-coding RNAs are typically prioritized, making mRNA enrichment the preferred method. The enriched mRNAs are often then reverse-transcribed into complementary DNA (cDNA) and sequenced using high-throughput platforms, such as Illumina^TM^, at a typical depth of 10 to 100 million reads per sample. The generated sequences undergo quality control assessment, including evaluation of Phred score (for base-calling accuracy), GC content (to detect contamination exogenous RNA), and fragment length distribution. After quality control, the reads are aligned to a reference genome to identify expressed genes. Gene expression levels are then quantified, normalized, and statistically compared to control samples to identify DEGs [189,223].

A key determinant of RNA-Seq data ratability is the number of biological replicates used per condition [223,224]. Empirical evidence suggests that a minimum of four replicates (samples) is required to ensure sufficient statistical power [224,225]. However, small sample sizes may limit the detection of druggable genes particularly in heterogeneous diseases such as HD. Phenotypic variability, both across individuals and disease stages, can obscure transcriptomic signals relevant to pathophysiology and therapeutic targeting.

Early transcriptomics studies in HD, based on limited postmortem material, suffered from small cohorts and incomplete clinical matching, which hampered reproducibility and consensus on therapeutic targets [187,188,191]. Notably, reanalysis by Dias-Pinto et al. [189] demonstrated that the lack of age matching between HD and control samples introduced systemic bias, resulting in false-negative and false-positive gene identification. These findings highlight the importance of careful cohort design and metadata control in transcriptomic studies of neurodegenerative disorders.

Beyond biological variation, technical aspects of differential expression analysis (DEA)—including statistical remodeling, normalization strategies, and multiple testing corrections—further influence target discovery [226,227,228]. To address these limitations and enhance the accuracy of therapeutic target identification, we developed BDASeq^®^, a tailored algorithm designed to reduce both type I and type II errors in RNA-Seq-based biomarker discovery [221,229].

BDASeq^®^ is an integrative analytics-based platform designed to facilitate identification of therapeutic targets through transcriptomic profiling. Central to its architecture is the Recursive Method Combination (RMC) framework, a novel ensemble strategy that aggregates the results of eight most widely used DEA algorithms (DESeq2 [228], edgeR [230], limma-Voom [231], NOISeq [232], EBSeq [233], dearseq [234], Wilcoxon [235], and Firth’s logistic regression [236]) [190]. This ensemble approach mitigates method-specific biases and improves the robustness of gene detection across heterogeneous datasets. Additionally, BDASeq integrates artificial intelligence (AI) algorithms that combine transcriptomic signature with clinical metadata, enabling prioritization of targets for CADD.

Leveraging BDASeq^®^, we conducted the largest transcriptomic meta-analysis in HD to date, reanalyzing 353 samples from 12 independent BioProjects (PRJNA271929, PRJNA316625, PRJNA670925, PRJEB59710, PRJNA253002, PRJNA557205, PRJNA602538, PRJNA531456, PRJNA398545, PRJNA729761, PRJNA388174, and PRJNA755746) deposited in the NCBI Sequence Read Archive. This large-scale integrative analysis identified 394 candidate druggable genes associated with HD pathogenesis [190]. This finding, which earned recognition at the MENA Congress for Rare Disease (Abu Dhabi, UAE, 16–19 May 2024) [221,222], opened new avenues for therapeutic development—particularly through drug repositioning strategies [190].

**Figure 6:**
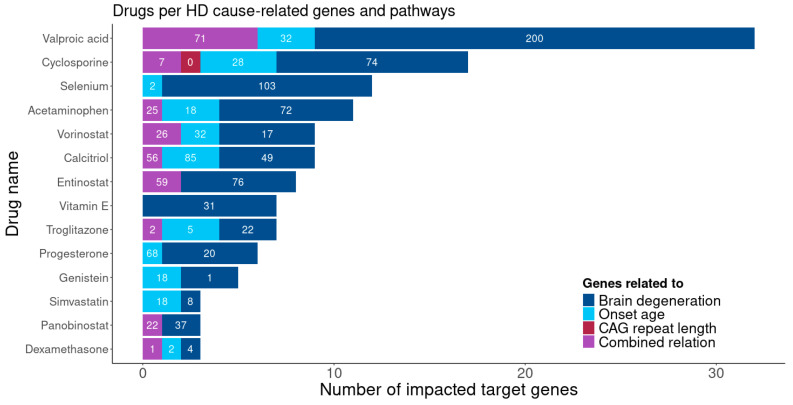
Candidate therapeutic compounds for HD identified through integrative AI-guided drug discovery. Drugs predicted using LBDD and pharmacogenomic approaches, based on 394 putative druggable genes identified by BDASeq^®^. Bar graph displays candidate compounds, with numbers within bars indicating the count of target genes modulated by each drug. This integrative strategy prioritizes compounds with potential to modulate multiple HD-relevant pathways, offering a platform for rational drug repurposing and multi-target intervention.

By integrating LBDD and pharmacogenomics approaches, several of the top-ranked gene targets identified by BDASeq^®^ have been linked to existing pharmacological agents with reported or putative activity in HD models. These include valproic acid, cyclosporine A, selenium, estradiol, acetaminophen, vorinostat, calcitriol, entinostat, vitamin E, troglitazone, progesterone, genistein, simvastatin, and Panobinostat and can offer new avenues for HD treatment (Figure 6). All these compounds have previously demonstrated potential neuroprotective, anti-inflammatory, or epigenetic effects relevant to HD, and their prioritization by BDASeq^®^ underscores the promise of AI-driven transcriptomics in accelerating drug repurposing for neurodegenerative diseases.

The listed compounds (Table 2) were prioritized based on their mechanistic relevance to HD pathophysiology—particularly targeting mitochondrial dysfunction, oxidative stress, neuroinflammation, and transcriptional dysregulation—and their potential for drug repositioning. For each drug, the table outlines the primary mechanism of action, specific HD-relevant effects observed in preclinical or clinical studies, the level of supporting evidence (ranging from in vitro models to clinical observations), and the corresponding literature references. Notably, several agents (e.g., valproic acid, cyclosporine A, genistein, and simvastatin) have shown the ability to cross the blood–brain barrier and ameliorate HD-related phenotypes in animal models or clinical settings. Others, such as entinostat, are emerging candidates requiring further validation. This comprehensive overview underscores the utility of BDASeq^®^ in uncovering actionable targets for HD and highlights both repurposed and novel compounds with translational potential.

Although these synthetic drugs target downstream symptoms or cellular stress responses, their clinical efficacy is often modest, inconsistent, or unproven in large-scale trials. While synthetic drugs remain essential for symptom management, their gene-specific action limits their ability to alter the natural history of HD. In contrast, cell-based therapies using human dental pulp stem-cell (hDPSC), such as NestaCell^®^, offer a multifaceted approach, as shown in Table 3. This is because the repertoire of biomolecules naturally produced by these cells, including BNDF, which confers neuroprotection, immunomodulation, and neuroregeneration, combined with their capability to cross the brain–blood barrier when intravenously administrated, positions NestaCell^®^ as a promising disease-modifying agent in the evolving landscape of HD therapeutics [7,211,213,215,269,270].

## 7. Conclusions

The landscape of HD treatment remains a significant clinical challenge, marked by the absence of effective disease-modifying therapies despite decades of intense research. While current pharmacological approaches may alleviate certain symptoms, particularly chorea in early-to-mid-stage HD, the overarching goal continues to be the development of interventions capable of altering the disease course. In this context, our study demonstrates the power of integrative transcriptomic analysis to overcome longstanding obstacles in target discovery. By developing BDASeq^®^, an AI-enhanced, ensemble-based differential expression analysis platform, we reanalyzed 353 samples from 12 independent BioProjects and identified 394 putative druggable genes linked to key pathological mechanisms in HD. However, to ensure the translational relevance of these targets, it is imperative to validate them using complementary methodologies. Techniques such as quantitative PCR (qPCR) and immunoassays can confirm gene and protein expression changes in patient-derived tissues. Furthermore, functional assays in cellular and animal models, including CRISPR-based gene editing and pharmacological modulation, are essential to establish the causal role of these targets in disease pathology. This enabled a systematic drug repositioning effort, nominating several FDA-approved compounds with mechanistic relevance and prior preclinical or clinical support. Our findings highlight the urgent need for innovative methodological frameworks in therapeutic discovery and clinical trial design. As the field moves forward, combining such transcriptomic insights with refined, biomarker-driven trials may finally usher in a new era of disease-modifying therapies for HD. A concerted, multidisciplinary effort will be essential to translate these promising avenues into tangible benefits for patients.

## Figures and Tables

**Table 1:** Comparative clinical outcomes of tetrabenazine and deutetrabenazine in HD.

	Direct Analyses ^1^		Indirect Analyses ^2^
Outcomes/Adverse Effects	TBZ-Placebo	DEU-Placebo	TBZ-DEU
UHDRS (chorea)	−3.20 ^3^	−2.50 ^4^	−1.00
UHDRS-TMS	−3.30	−4.00 ^4^	0.70
Depression scale	0.76 ^5^	−0.18 ^4^	0.94
Insomnia scale	1.80 ^5^	−0.30 ^4^	2.10
Severe adverse effects	5.44	1.00	5.44
Drowsiness	13.32 ^3^	2.69	4.95
Diarrhea	0.72	9.87	0.07
Insomnia	21.84 ^3^	1.54	14.18
Fatigue	1.86	1.54	1.21
Fall	1.30	0.48	2.71
Depression	11.15	0.65	17.15

^1^ For direct analyses, values presented show the difference in mean score between those treated with the drug under investigation and placebo. Positive values favor placebo and negative values favor treatment. ^2^ For indirect analyses, values represent differences in mean score between tetrabenazine and deutetrabenazine. Positive values favor deutetrabenazine and negative values favor tetrabenazine. ^3^ Significantly favorable for tetrabenazine. ^4^ Significantly favorable for deutetrabenazine. ^5^ Significantly favorable for placebo. Source: modified from Rodrigues et al. [204].

**Table 2:** Summary of candidate therapeutic agents for HD identified via BDASeq^®^.

Drug	Mechanism of Action	HD-Relevant Effects	Evidence Level	References
Valproic acid	GABAergic activity; HDAC inhibition	Reduces aggression, improves motor symptoms (e.g., myoclonic hyperkinesia); potential mood stabilizer	Preclinical ^1^ & clinical ^2^	[237,238,239]
Cyclosporine A	Calcineurin inhibition; mitochondrial protection	Attenuates mitochondrial dysfunction and oxidative stress; improves behavior in HD models	Preclinical	[240,241]
Selenium	Antioxidant; glutathione peroxidase cofactor	Addresses selenium deficiency in HD brains; reduces oxidative damage	Preclinical and Post-mortem Human ^3^	[242,243,244]
Estradiol	Estrogen receptor activation; mitochondrial regulation	Reduces oxidative stress and inflammation; modulates energy metabolism	Preclinical	[245,246,247]
Progesterone	Neurosteroid; anti-inflammatory	Reduces oxidative stress; neuroprotective in HD models	Preclinical	[247,248,249,250]
Acetaminophen	COX inhibition; analgesic	Pain relief for muscle aches in early-stage HD	Clinical Use (Non-HD-specific)	[251,252]
Panobinostat	HDAC inhibition	Modulates gene expression; potential to reduce neurodegeneration	Preclinical	[253]
Vorinostat (SAHA)	HDAC inhibition	Improves neuronal survival; reduces oxidative stress	Preclinical	[254,255,256]
Entinostat	HDAC1/3 selective inhibitor	Putative neuroprotective, but not yet studied in HD models	Theoretical ^4^	—
Calcitriol	Vitamin D receptor activation	Reduces oxidative stress, inflammation; modulates calcium homeostasis and mitochondria	Preclinical	[257,258]
Vitamin E	Antioxidant	Protects neurons from oxidative stress and lipid peroxidation	Preclinical and Clinical (limited)	[259,260]
Troglitazone	PPAR-γ agonist	Reduces oxidative stress; improves mitochondrial and energy metabolism	Preclinical	[261]
Genistein	Autophagy induction; estrogenic activity	Clears mHTT aggregates; improves motor, cognitive and behavioral outcomes	Preclinical	[262,263,264,265]
Simvastatin	Cholesterol modulation; anti-inflammatory	Crosses BBB; reduces oxidative stress; potential neuroprotection	Preclinical and Observational	[58,266,267]
Dexamethasone	Glucocorticoid receptor agonist; heat shock response	Reduces mHTT aggregation; improves behavioral phenotype	Preclinical	[268]

^1^ Preclinical: evidence derived from in vitro or animal models. ^2^ Clinical: evidence from human trials or patient treatment studies. ^3^ Postmortem human: observations from postmortem tissue. ^4^ Theoretical: hypothetical or inferred based on drug class, not yet experimentally tested in HD.

**Table 3:** Main differences between synthetic drugs and hDPSC-based treatments.

Feature	Synthetic Drugs	hDPSC-Based Treatments
Targets	Narrow (few genes or pathways)	Broad (multi-pathway modulation)
Neuroprotection	Limited	Strong (via trophic support)
Neuroregeneration	Absent	Potential for neuronal replacement
Immunomodulatory action	Indirect	Direct immunomodulatory effects
Disease modification	Unlikely	Promising in preclinical models and Phase I/II clinical trial

## Abbreviations

The following abbreviations are used in this manuscript:

ABH	Brazil Huntington Association
BDASeq^®^	Biomarker Discovery Algorithm
CADD	Computer-aided drug design
CAG	Cytosine–adenine–guanine
DEA	Differential expression analysis
DEG	Differentially expressed genes
HD	Huntington’s disease
RMC	Recursive Method Combination
TFC	Total Function Capacity
TMS	Total Motor Score
UHDRS	Unified Huntington’s Disease Rate Scale
